# Immunomodulatory streptococci that inhibit CXCL8 secretion and NFκB activation are common members of the oral microbiota

**DOI:** 10.1099/jmm.0.001329

**Published:** 2021-03-18

**Authors:** Sarah Myers, Thuy Do, Josephine L. Meade, Aradhna Tugnait, Jon J. Vernon, Jelena Pistolic, Robert E. W. Hancock, Philip D. Marsh, Harsh M. Trivedi, Dandan Chen, Deirdre A. Devine

**Affiliations:** ^1^​ Division of Oral Biology, School of Dentistry, University of Leeds, Leeds, UK; ^2^​ Division of Restorative Dentistry, University of Leeds, Leeds, UK; ^3^​ Centre for Microbial Diseases and Immunity Research, University of British Columbia, Columbia, Canada; ^4^​ Colgate Palmolive Inc., NJ, USA

**Keywords:** CXCL8, gingivitis, immunomodulation, oral microbiology, streptococci

## Abstract

**Introduction:**

Oral tissues are generally homeostatic despite exposure to many potential inflammatory agents including the resident microbiota. This requires the balancing of inflammation by regulatory mechanisms and/or anti-inflammatory commensal bacteria. Thus, the levels of anti-inflammatory commensal bacteria in resident populations may be critical in maintaining this homeostatic balance.

**Hypothesis/Gap Statement:**

The incidence of immunosuppressive streptococci in the oral cavity is not well established. Determining the proportion of these organisms and the mechanisms involved may help to understand host-microbe homeostasis and inform development of probiotics or prebiotics in the maintenance of oral health.

**Aim:**

To determine the incidence and potential modes of action of immunosuppressive capacity in resident oral streptococci.

**Methodology:**

Supragingival plaque was collected from five healthy participants and supragingival and subgingival plaque from five with gingivitis. Twenty streptococci from each sample were co-cultured with epithelial cells±flagellin or LL-37. CXCL8 secretion was detected by ELISA, induction of cytotoxicity in human epithelial cells by lactate dehydrogenase release and NFκB-activation using a reporter cell line. Bacterial identification was achieved through partial 16S rRNA gene sequencing and next-generation sequencing.

**Results:**

CXCL8 secretion was inhibited by 94/300 isolates. Immunosuppressive isolates were detected in supragingival plaque from healthy (4/5) and gingivitis (4/5) samples, and in 2/5 subgingival (gingivitis) plaque samples. Most were *Streptococcus mitis/oralis*. Seventeen representative immunosuppressive isolates all inhibited NFκB activation. The immunosuppressive mechanism was strain specific, often mediated by ultra-violet light-labile factors, whilst bacterial viability was essential in certain species.

**Conclusion:**

Many streptococci isolated from plaque suppressed epithelial cell CXCL8 secretion, via inhibition of NFκB. This phenomenon may play an important role in oral host-microbe homeostasis.

## Introduction

Many human tissues are colonized by bacterial communities [1] that form a complex microbiota that is unique to each individual. In health, such tissues do not normally enter a state of irreversibly damaging inflammation and retain the ability to respond adequately to pathogenic challenges. This host-microbe balance is maintained by homeostatic mechanisms that are proposed to include regulation or modulation of host responses by commensal organisms. Resident microbial communities contain complex mixtures of bacteria, some of which elicit little or no host response, some that promote pro-inflammatory responses, and others that are actively anti-inflammatory [2–5], but they have coevolved with their host species, and have helped to establish a threshold of defensive tissue activation required for immune fitness [6]. Body compartments like the gut and oral cavity are colonized by complex microbiota that contain microbial signature molecules such as lipopolysaccharide, lipoteichoic acid and flagellin, which are recognized by and can activate the innate immune system. However, rather than causing inflammation in these tissues, there is a situation of homeostasis whereby prospective inflammation is likely balanced by regulatory mechanisms and the activities of anti-inflammatory members of the microbiota [4]. These beneficial commensal organisms, therefore, have been proposed by certain authors to be key in maintaining healthy gut tissues that are primed to respond rapidly to pathogens, but are protected from being damaged by inflammatory responses to the indigenous/resident microbiota [7, 8]. Conversely, other researchers have hypothesized that the widespread possession of anti-inflammatory ability by resident gut mucosal bacteria could be detrimental, by imposing an unsustainable burden on the host immune system and compromising its ability to respond appropriately to pathogens [9].

Loss of the balance between resident populations and the host response to them (dysbiosis) contributes to the incidence of some significant, multifactorial diseases [8, 10]. Periodontal diseases are examples of dysbioses, since they are caused by changes in the balance and composition of resident plaque microbial communities and a subsequent loss of host-microbe homeostasis. The failure of the immune system to limit the microbial community and resultant exaggerated and subverted local host immune response can result in tissue damage [11, 12]. Healthy gingival tissues, like the gut, are heavily colonized and the balance of pro- and anti-inflammatory resident microorganisms may play similar roles to those proposed to be important in the gut in maintaining healthy tissues and preventing tipping of the balance to dysbiosis. Suppression of inflammatory mediator secretion is emerging as a property of some oral commensal streptococci [4, 13–18], but the prevalence of such anti-inflammatory organisms in the mouth is unknown. This study aimed to examine their potential roles in oral host-microbe homeostasis by determining if immunosuppressive properties are common amongst resident oral streptococci from sites classed as being healthy or displaying mild gingivitis.

## Methods

### Subject selection and sample collection

**Fig. 1.**
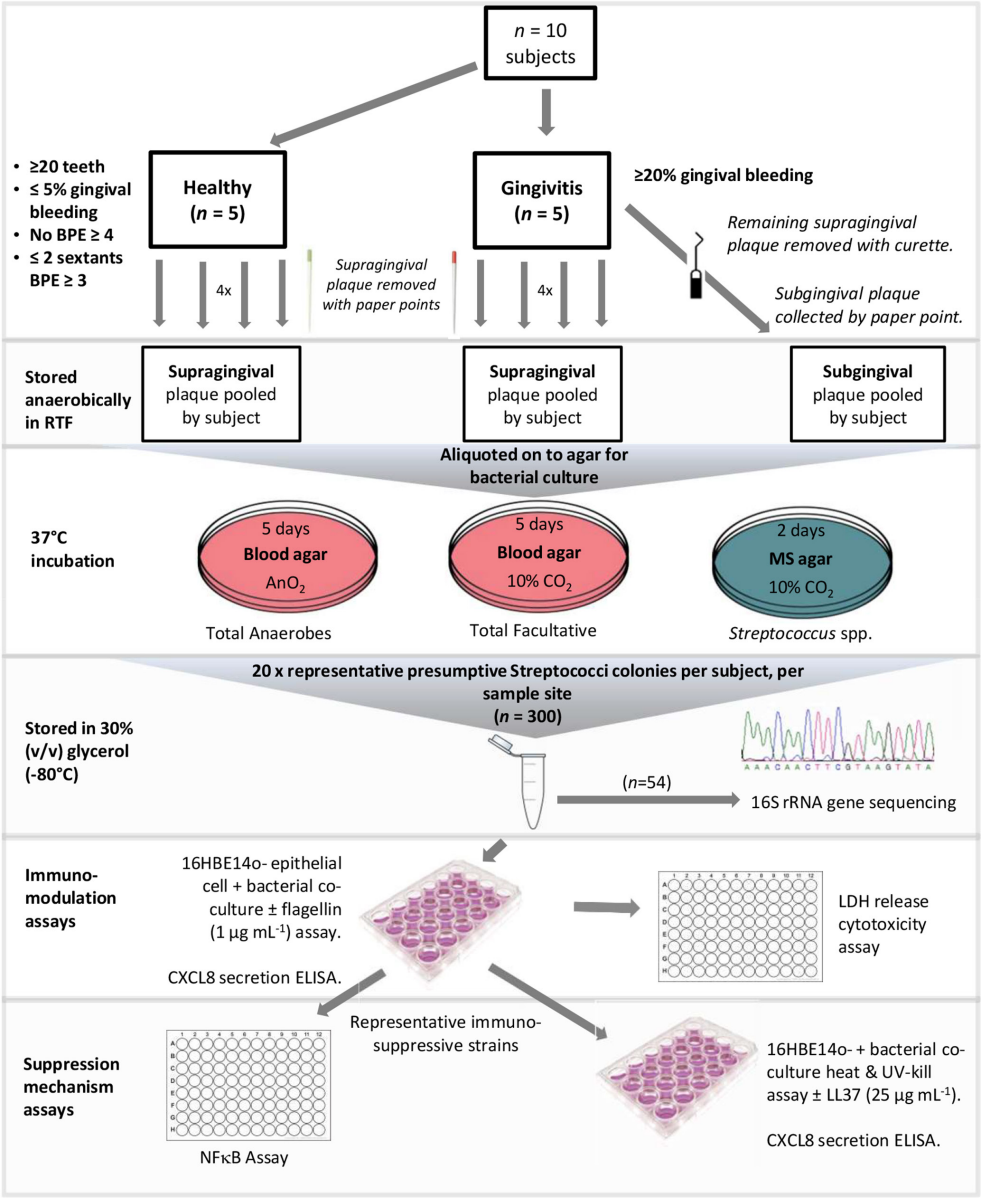
Flow chart of study design and methodology. MS – mitis salivarius, RTF – reduced transport fluid, LDH – lactate dehydrogenase, BPE – basic periodontal examination.

Study design and methodologies are illustrated in Fig. 1. Volunteers were required to have no active caries, a low history of caries and had not taken a course of antibiotics in the previous 6 months. Subjects were recruited to the healthy control group (*n*=5) if they had 20 or more teeth and gingival bleeding at up to 5 % of gingival sites with no sextant of the mouth recording a Basic Periodontal Examination (BPE) code of four and no more than two sextants recording a BPE code of three. If they had gingival bleeding of 20 % or more sites, they were placed in the gingivitis group (*n*=5). Four of the five subjects recruited to the control group (age range 23–53 years; mean 35 years) were female; as were three of the gingivitis group (26–30 years; mean 26.8 years). Pooled supragingival plaque samples from each participant, taken with sterile paper points from four clinically healthy sites in the healthy group and from four diseased sites in the gingivitis group, were placed into 1 ml pre-reduced transport fluid (RTF) [19] containing 1 mm glass beads. Samples were retained in an anaerobic cabinet (Don Whitley Mark III, Otley, UK) in an atmosphere of 10 % CO_2_, 10 % H_2_ and 80 % N_2_. Additionally, for the gingivitis group, the remaining supragingival plaque at each site was removed using a sterile curette and subgingival plaque was collected with sterile paper points and pooled in RTF. Preliminary experiments also included sampling the tongue of one orally healthy individual, by scraping with a sterile swab.

### Bacterial isolation and identification

Viable bacterial counts of plaque samples were determined by serial dilution in RTF and inoculation onto pre-reduced blood agar (BA) plates (Oxoid, UK) followed by 5 days incubation at 37 °C anaerobically (total counts) or in 10 % CO_2_ (total facultative counts). Mitis Salivarius agar (MSA; Fluka, UK) incubated for 2 days at 37 °C in 10 % CO_2_ was used to enumerate *Streptococcus* populations in plaque samples.

Twenty representative presumptive streptococci from each original sample were isolated. To do this, representative colonies of each morphological type on MSA were collected in proportion to their numbers on the isolation plate (e.g. type-1 colony accounted for 50 % of total streptococcal count so 10 colonies picked; type-2 colonies represented 25 % of the total so five colonies picked). They were Gram-stained, streaked onto fresh MSA plates and were stored in 30 % (v/v) glycerol at −80 °C until required for further analysis. To identify streptococcal isolates, a bacterial suspension in sterile water was heated at 100 °C for 10 min to extract the DNA. Samples were centrifuged at 13 000 r.p.m. for 30 s and the supernatant used as a template for amplification of partial 16S rRNA genes using the universal primers 27 f and 1492 r. Amplicons were sequenced using 519 r and a BigDye terminator cycle sequencing kit (Thermofisher, UK), with a 3730xl DNA analyser (Applied Biosystems, UK). Additional species confirmation was obtained through next-generation sequencing (NGS) using an Illumina HiSeq3000 sequencing system. Sequences were subjected to blast searching (http://www.ncbi.nlm.nih.gov/blast/Blast.cgi) to identify the isolates and phylogenetic dendrograms were constructed using the Neighbour-Joining algorithm, in mega version5 (http://www.megasoftware.net/).

### CXCL8 secretion by epithelial cells

The SV40-transformed, immortalized human bronchial epithelial cell line 16HBE14o- (gifted by Dr D. Gruenert; University of California, USA) were cultured, passaged and seeded into 24-well plates at a density of 10^5^ cells/well, as described by Cosseau *et al*. [4]. Complete MEM was removed from the monolayers (ca 95 % confluent) and replaced by serum-free MEM; following 2 h incubation, the monolayers were stimulated with 1 µg ml^−1^ flagellin (Sigma, UK)±co-incubation with streptococci at a m.o.i. of 50 : 1. In some experiments flagellin was replaced by 25 µg ml^−1^ cathelicidin LL37 (Pepceuticals, UK) or 25 µg ml^−1^ polyinosinic:polycytidylic acid (poly I:C, Sigma). Streptococci were prepared by growing overnight in brain heart infusion broth (BHI; Oxoid) in 10 % CO_2_ at 37 °C and washing in tissue culture grade phosphate buffered saline (PBS; Sigma; pH 7.4). In some cases, streptococci in PBS were killed prior to addition to epithelial cells by heating at 100 °C for 15 min in a benchtop heat block (Techne Dri block DB 2A; Jencons Scientific, UK) or by pulsed exposure (3×10 min, 250 mJ cycles) to ultraviolet light (UV; UV Stratalinker 2400). After co-incubation for 24 h, cell culture supernatants were centrifuged at 7000 ***g*** for 5 min to remove bacteria and cell debris. As a measure of cytotoxicity, lactate dehydrogenase (LDH) in tissue culture supernatants was assayed in triplicate using a CytoTox 96 Non-radioactive Cytotoxicity assay (Promega Corp, Madison, WI, USA). CXCL8 was detected using commercially available ELISA antibody pairs and kit (R and D Research Systems UK) according to the manufacturer’s instructions.

### Activation of NFκB

The stable A549/NFκB-luc cell line (Panomics P/N LR0051; Fremont, CA) was grown in Dulbecco’s modified Eagle's medium (DMEM) containing 10 % (v/v) foetal bovine serum, 2 mM l-glutamine, and 100 μg ml^−1^ hygromycin at 37 °C in a humidified 5 % CO_2_ incubator. 5×10^4^ cells were seeded per well of a 96-well plate and grown overnight. Following the addition of 50 μg ml^−1^ tumour necrosis factor (TNF)-α±streptococci at a m.o.i. of 50 : 1 or 10 : 1, monolayers were incubated for 6 h. Luciferase activity was measured with a Bright-Glo luciferase assay kit (Promega); following the manufacturer’s instructions and using a Thermo Scientific Varioskan Flash plate reader at wavelengths of 550 nm. Negative controls comprised the A549/NFκB-luc cells alone and positive controls consisted of cells plus TNF-α. The NFκB inhibitor BAY 11–7085 (40 μg ml^−1^) was used to confirm NFκB activation by TNF-α.

## Results

### Suppression of CXCL8 secretion was a property of many resident oral streptococci

**Fig. 2.**
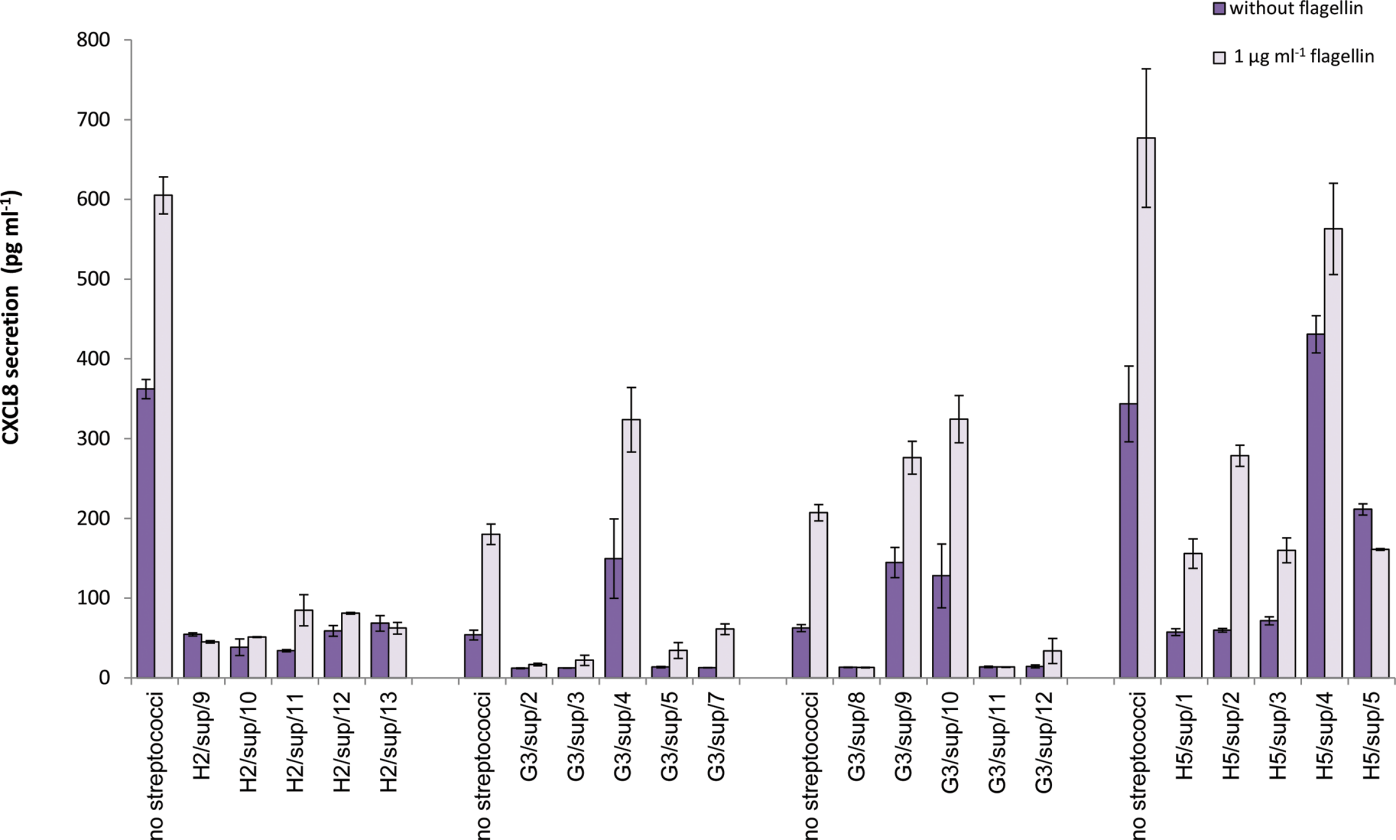
CXCL8 secretion by 16HBE14o- cells incubated with streptococci isolated from plaque in the presence and absence of 1 µg ml^−1^ flagellin. Results are shown from four representative co-culture assays performed using a selection of streptococci isolated from the supragingival (sup) plaque of members of the healthy (**H2 and H5**) or gingivitis (**G3**) group. The data series ‘without flagellin’ reflects unstimulated/baseline secretion. Error bars represent±standard error of the mean.

**Fig. 3.**
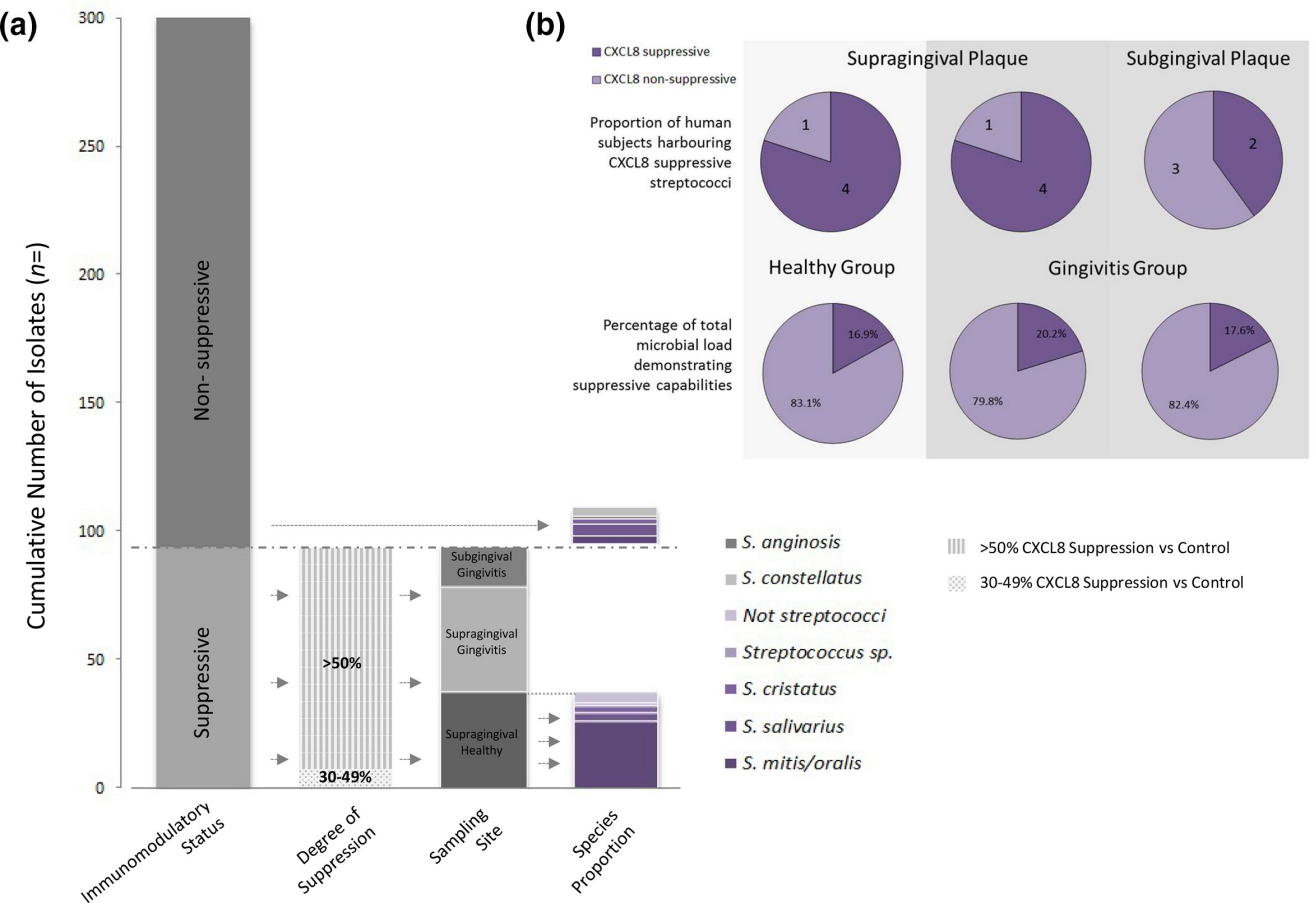
(a) Distribution of bacterial isolates from plaque by immunomodulatory status. In total, 94 suppressive isolates delineated by degree of suppression → sampling site → species. Immunomodulatory capability determined by CXCL8 suppression in co-culture with 16HBE14o- cells±stimulation with 1 µg ml^−1^ flagellin. Bacterial identification by partial 16 s rRNA or next-generation sequencing. Only representative samples were subjected to molecular species identification. (b) Proportions of test groups/sampling sites demonstrating CXCL8 suppression by co-culture assay (top). Mean calculated prevalence of total microbial loads with CXCL8 suppressive capabilities. Suppressive isolate prevalence ranged between 3–47 % (bottom).

We previously showed that *Streptococcus salivarius* K12 inhibited bacterial, flagellin and LL37-stimulated CXCL8 secretion by bronchial, skin and oral primary cells and cell lines [4], and subsequently that K12 was able to suppress CXCL8 secretion by an oral keratinocyte cell line in response to oral bacteria; (Fig. S1, available in the online version of this article). Furthermore, we have demonstrated substantial reductions in CXCL8 release by the same cell line, with the addition of K12 to multi-species co-cultures of *Porphyromonas gingivalis */* Fusobacterium nucleatum* and *Aggregatibacter actinomycetemcomitans/F. nucleatum* resulting in decreases of 68.9 and 59.8 %, respectively (unpublished data). Since K12 was originally isolated from the tongue; in preliminary experiments we isolated streptococci from the tongue of one individual. Of the 21 representative tongue streptococci isolated, nine inhibited CXCL8 secretion from 16HBE14o- cells stimulated with 1 µg ml^−1^ flagellin, 25 µg ml^−1^ LL-37 or poly-I:C; (Fig. S2). This indicated that the ability to suppress CXCL8 responses may be relatively common in resident oral streptococci, and that examination of 20 isolates from each sample would be sufficient to detect the incidence of immunosuppressive streptococci in plaque. A total of 300 presumptive streptococci were isolated and co-incubated with 16HBE14o- cells and flagellin. Of these, 31.3 % (94/300) were defined as immunosuppressive, reducing flagellin-stimulated CXCL8 secretion by at least 30 % (representative data are shown in Fig. 2); 86 of these 94 reduced flagellin-induced CXCL8 secretion by ≥50 %, and most also reduced baseline/unstimulated CXCL8 secretion. Distributions of isolates by immunomodulatory status are illustrated in Fig. 3, Table 1. In cases where CXCL8-suppressive isolates were detected, their incidences in plaque were calculated to range from approximately 3–47 % of the total cultivable microbial load; with mean incidences of 16.9, 20.2 and 17.6 % detected for healthy supragingival, gingivitis supragingival and subgingival plaque, respectively. No significant differences were observed (Mann–Whitney and Wilcoxon signed ranks tests). Plaque sample viable counts are displayed in Table S1.

**Table 1.** Incidence of immunosuppressive streptococci in plaque samples from healthy and gingivitis subjects

Subject	Healthy/gingivitis	Plaque	No. immuno-suppressive isolates (%)
H1	Healthy	Supragingival	3 (15)
H2	Healthy	Supragingival	17 (85)
H3	Healthy	Supragingival	0 (0)
H4	Healthy	Supragingival	4 (0)
H5	Healthy	Supragingival	13 (65)
**Total**			**37/100**
G1	Gingivitis	Supragingival	9 (45)
G2	Gingivitis	Supragingival	0 (0)
G3	Gingivitis	Supragingival	11 (55)
G4	Gingivitis	Supragingival	9 (45)
G5	Gingivitis	Supragingival	12 (60)
**Total**			**41/100**
G1	Gingivitis	Subgingival	0 (0)
G2	Gingivitis	Subgingival	0 (0)
G3	Gingivitis	Subgingival	8 (40)
G4	Gingivitis	Subgingival	0 (0)
G5	Gingivitis	Subgingival	8 (40)
**Total**			**16/100**

In the absence of streptococci, the mean LDH release was 8.4 % (±5.6) and 7.9 % (±3.8) of the cell death control for 16HBE14o- cells alone and stimulated with flagellin, respectively. Streptococci caused a modest (7.3 %; *P* <0.01) increase in mean LDH release compared with the streptococci-free controls; a small proportion (5 %) caused >30 % LDH release and were considered cytotoxic.

### Resident streptococci representing a range of species inhibited CXCL8 secretion

Preliminary investigations of 21 streptococci from the tongue of one individual identified 13 *S *. *salivarius* and six *Streptococcus parasanguinis* isolates, of which seven and two were determined as CXCL8-suppressive, respectively. A further two isolates that were classified as *Streptococcus cristatus *, demonstrated high levels of cytotoxicity to 16HBE14o- cells according to the LDH release assay.

A panel of isolates were selected for species identification (*n*=54), using partial 16S rRNA gene sequencing and confirmed with NGS where necessary. Due to a probiotic focus of research interests, these encompassed all of the immunosuppressive strains and representative non-suppressive isolates from healthy supragingival plaque (*n*=41), along with multiple (*n*=13) representative suppressive and non-suppressive isolates from both sub- and supragingival sites of gingivitis subjects (Table 2). The majority of CXCL8-suppressive streptococci from plaque were identified as *Streptococcus mitis/oralis*, whilst the 13 non-suppressive isolates included representatives of five species, distributed relatively evenly. There was a tendency for isolates from the same subject to be clustered together in phylogenetic dendrograms and there were examples of apparently closely related isolates differing in terms of CXCL8-suppressive ability; (Fig. S3).

**Table 2.** Identification of representative streptococci displaying either immunosuppressive (*n*=41) or non-immunosuppressive (*n*=13) properties

Suppressive/non-suppressive	Species	No./total identified	From subjects
Non-suppressive	* S. anginosus *	1/13	H1
	* S. constellatus *	3/13	H1, H2, G1
	* S. cristatus *	2/13	H1, G3
	*S. mitis/oralis*	3/13	H2, H4, G3
	* S. salivarius *	4/13	H2, H3, H4, G1
Suppressive	* S. cristatus *	3/41	G1, G3
	*S. mitis/oralis*	34/41	H1, H2, H4, H5, G3, G4
	* S. salivarius *	3/41	H2, H4
	* Streptococcus* sp*.2_1_36FAA*	1/41	H4

### CXCL8-suppressive isolates inhibited NFκB activation

**Fig. 4.**
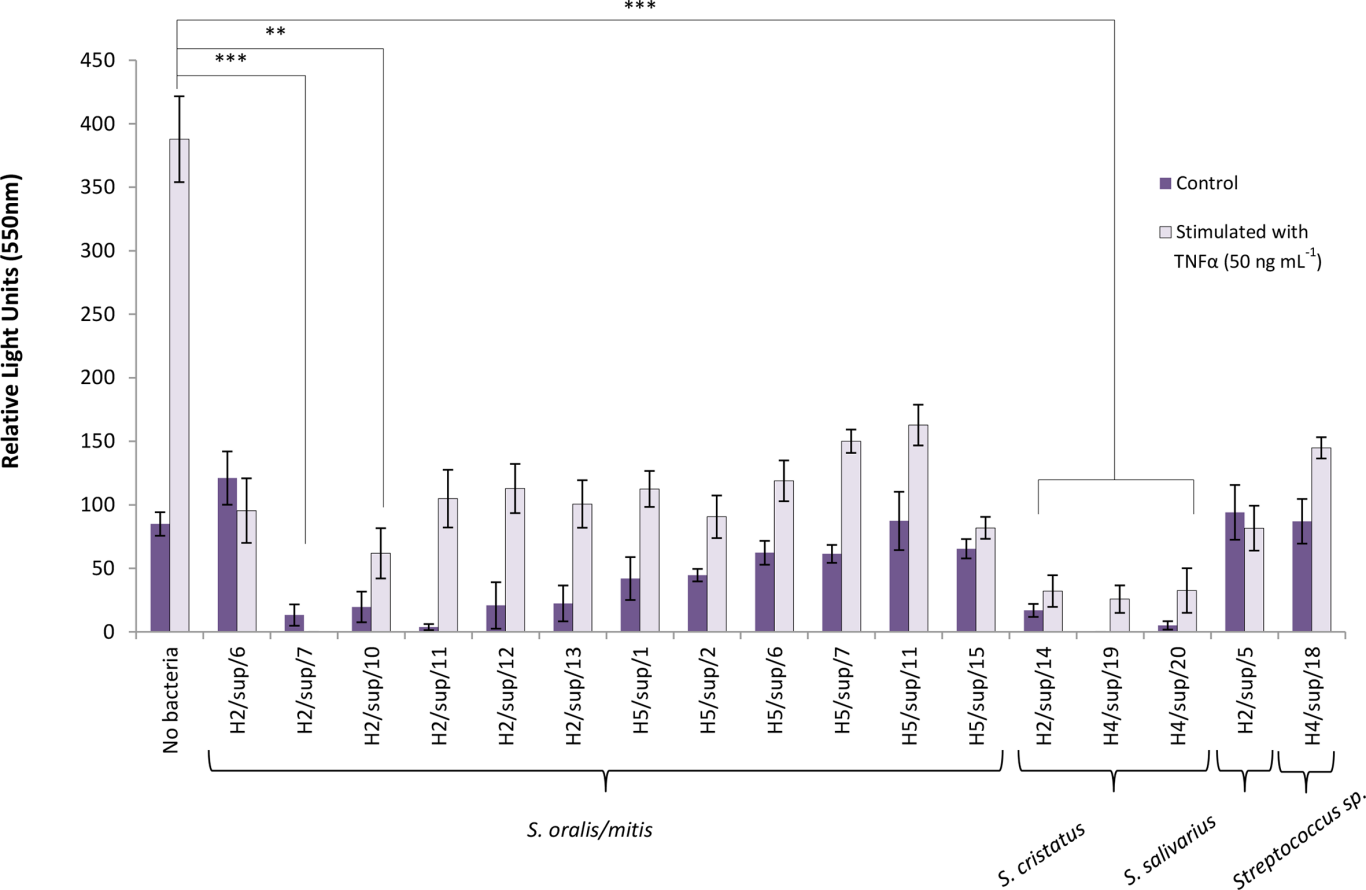
Inhibition of NFκB activation in A549/NFκB-luc cell line by (*n*=17) immunomodulatory streptococci isolated from plaque (multiplicities of infection 50 : 1) following 6 h incubation. Species designations were based on blast comparisons of partial 16S rRNA gene sequences. Error bars represent ±standard error of the mean. To evaluate whether samples originated from the same distribution, a Kruskal–Wallis test was performed; *P* <0.001. To assess the variance between individual pairs of groups a Mann–Whitney U post-hoc test with Bonferroni correction was used. Adjusted *P*-values are displayed *P ≤0.05, ***P <0.001.

Luciferase expression was induced in the A549/NFκB-luc cell line when stimulated with TNF-α, but this was significantly suppressed by the addition of the majority (*n*=15/17) of representative CXCL8-suppressive streptococci strains isolated from plaque; (Fig. 4). *S. salivarius* S16 and *S. parasanguinis* S25, isolated from the tongue, also significantly inhibited NFκB secretion by this reporter cell line (data not shown).

### CXCL8-suppression could be mediated by multiple mechanisms

**Fig. 5.**
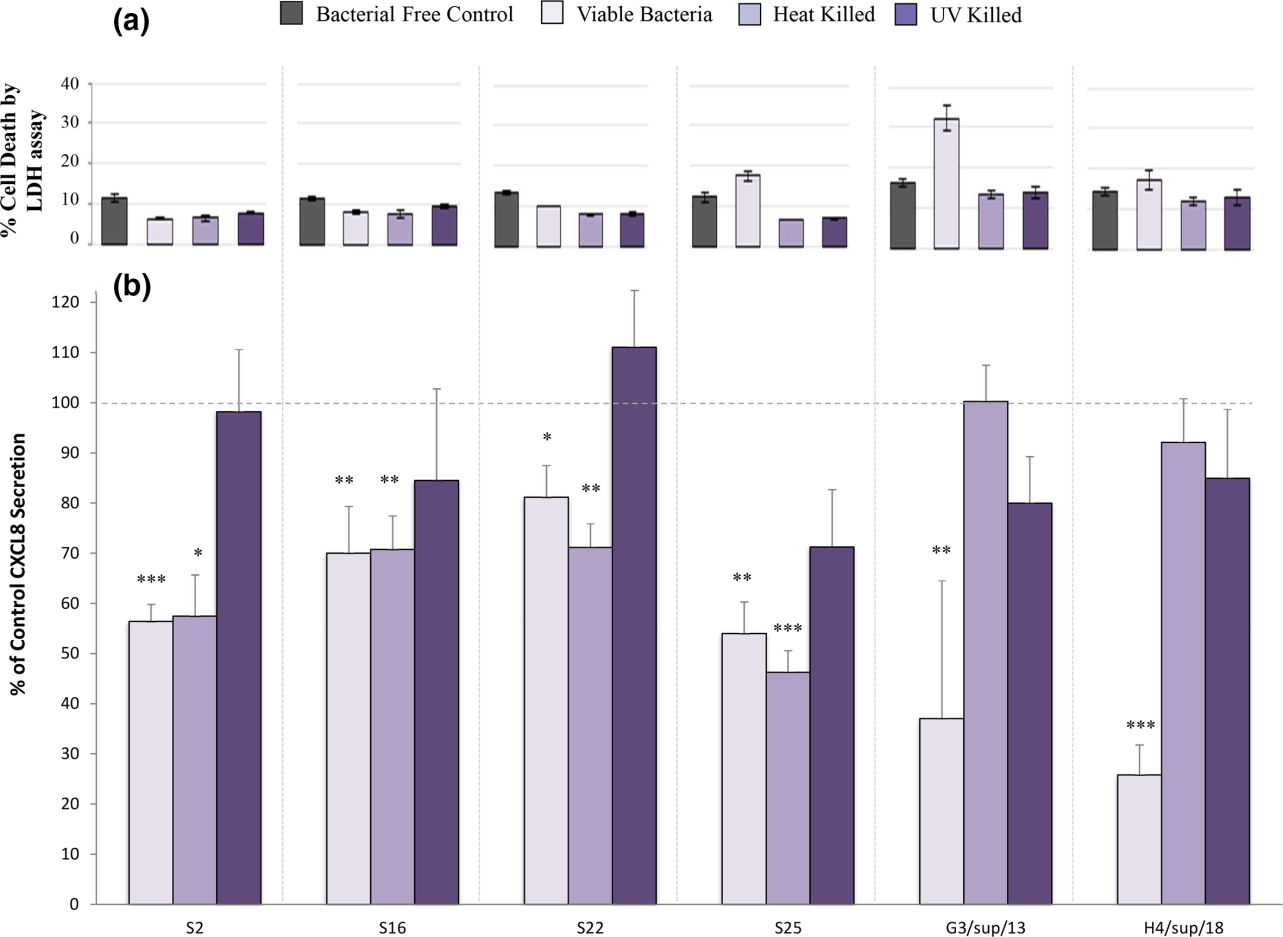
Effect on epithelial cell secretion of CXCL8 of treatment with live, heat-killed and ultra-violet light killed (UV) *Streptococcus salivarius* (**S2, S16**) *Streptococcus parasanguinis* (**S22, S25**), *Streptococcus mitis/oralis* (G3/sup/13) and *Streptococcus sp.2_1_36FAA* (H4/sup/18). (a) Percentage of 16HBE14o- epithelial cell death, proportional to 100 % cell death control, assessed by the lactate dehydrogenase (LDH) assay to determine the cytotoxic effect of the bacteria. (b) CXCL8 secretion by LL37-stimulated 16HBE14o- cells, expressed as a percentage of bacterial free controls, after treatment with representative tongue and plaque streptococci. Statistical significance refers to comparisons with LL-37-stimulated control cell secretion (in the absence of bacteria) *=p <0.05; **=p <0.01; ***=p <0.001. Error bars represent the standard error of the mean.

We determined the dependence of immunomodulation on bacterial viability to offer insight into possible suppressive mechanisms. *S. salivarius* S2 and S16, as well as *S. parasanguinis* strains S22 and S25, continued to inhibit CXCL8 secretion after heat-killing, but not after being killed by exposure to UV light. In contrast, both heat- and UV-killing of representative strains of *S. mitis/oralis* and *Streptococcus* sp. *2_1_36FAA* resulted in CXCL8 secretion comparable to 16HBE14o- cells alone (Fig. 5).

## Discussion

Immunosuppressive capabilities have been described for an increasing range of commensal or probiotic organisms, but studies have not been undertaken to determine the prevalence of such properties in resident microbial communities. Streptococci are significant members of the healthy resident oral microbiome. This study has demonstrated that more than 30 % of the streptococci isolated from supragingival and subgingival plaque from ten human volunteers were able to suppress the secretion of CXCL8 by cultured epithelial cells. Thus, this ability appears to be relatively common in resident streptococci *in vitro* and has the potential, therefore, to play a role in the maintenance of host-microbe homeostasis in the mouth, or in loss of homeostasis if such organisms are present in numbers that are too high. It is likely that the relative balance of pro-inflammatory and immunosuppressive resident organisms is critical for appropriate immune responses and the maintenance of host-microbe homeostasis. While some plaque samples appeared to harbour potentially immunosuppressive streptococci in numbers too low to be detected in 20 representative isolates, in other supragingival plaque samples they appear to have accounted for a substantial proportion of the total cultivable microbial load. There were no significant differences in occurrence of immunosuppressive streptococci comparing the healthy and gingivitis groups, although larger numbers of subjects will be required to confirm or contradict this and should include individuals with periodontitis (where more pronounced dysbioses would be expected), as well as gingivitis. *S. salivarius* K12 suppressed CXCL8 secretion from all non-oral and oral cell types we examined, and therefore 16HBE14o- cells were used as a well-characterized screening tool. The isolates from this study were shown to be akin to K12 in inhibiting NFκB activation, but it will be essential to confirm that they behave in a similar way with other epithelial cells, including those of oral origin. Other commensal bacteria should also be considered, along with a broader characterization of the inflammatory response to investigate other pro-inflammatory pathways that might be manipulated by such species. For example, *Weissella cibaria *, an organism that has been associated with periodontal health [20], inhibited CXCL8 and CXCL6 secretion by human oral epithelial, KB cells in response to stimulation by *F. nucleatum* [21]. Furthermore, induction of the nicotinic acetylcholine pathway in *S. salivarius* treated cells [4], suggests that investigations into stimulatory effects on anti-inflammatory pathways may also prove valuable.

The ability to suppress CXCL8 secretion *in vitro* was displayed by streptococci belonging to a range of species (*S. mitis/oralis*, S. *salivarius *, *S. parasanguinis *, *S. cristatus *, *S. anginosus *, *S. constellatus *), but in both supragingival and subgingival plaque this property was predominantly associated with *S. mitis/oralis*. Since, subgingival plaque is derived from supragingival plaque that spreads down into the gingival sulcus [22, 23], we might expect to see a correlation of immunomodulatory species. The pioneer species that colonize teeth are mainly streptococci, particularly *S. mitis* and *S. oralis,* with smaller proportions of *Actinomyces* and other species. *S. mitis* and *S. oralis* are commonly isolated from dental plaque and *S. mitis* also often associates with mucosal sites [23, 24]. Both species are members of a group of organisms that have been proposed to be ‘true’ oral commensals [25]. Belde-ferra *et al*. (2012) have proposed that strains of *S. oralis, S. mitis* and *Streptococcus sanguinis* are potential oral probiotics, since they are not only associated with oral health, but can also antagonize cariogenic streptococci [26]. On the other hand, *S. mitis* has been associated with opportunistic infections outside the oral cavity [27].

Certain commensal streptococci encompassing representatives of *S. salivarius, Streptococcus vestibularis* and *S. cristatus* have been described in previous studies as immunosuppressive in a range of cell types *in vitro*, and *S. salivarius* inhibits inflammation in an animal model [4, 13–17], as well as in humans in preventing recurrent pharyngitis and/or tonsillitis caused by *Streptococcus pyogenes *, and recurrent acute otitis media [28]. The roles of such immunosuppressive organisms in host-microbe homeostasis *in vivo* in the mouth is not known; however, Twetman *et al.* demonstrated that the use of chewing gum containing immunosuppressive probiotic lactobacilli was associated with transient reductions in CXCL8 secretion into gingival crevicular fluid [29].

Here, we demonstrated immune suppression of cultured epithelial cells by mono-cultures of streptococci. While the primary purpose of this study was to investigate how common this property may be in oral streptococci, it is important to recognize that *in vivo* these bacteria are components of complex communities. Our early experiments indicated K12 was effective at inhibiting responses to individual species and co-aggregating suspensions of *P. gingivalis* or *A. actinomycetemcomitans* and *F. nucleatum* (Fig. S1 and unpublished data). However, studies assessing the immunological impact of oral biofilms have demonstrated contrasting responses between single- and multi-species models [30, 31]. It is clear that there is considerable variation in both single and multi-species models in terms of the responses induced by different species of streptococci as demonstrated, for example, by our data and that of Belibasakis *et al.*, which highlighted an upregulation of IL8 production associated with *Streptococcus gordonii* and *S. oralis* and a down-regulation associated with *S. sanguinis* [30]. Our study further indicates strain-dependent immunosuppressive abilities within a species.

A range of mechanisms have been described whereby commensal organisms inhibit or suppress inflammatory responses, including modulation of Toll-like receptor or NOD-like receptor expression and signalling, inhibition of activation of NFκB or increasing the secretion of anti-inflammatory cytokines [4, 8, 32]. Inhibition of NFκB activation has been proposed to underlie the immunosuppression observed in other studies of commensal streptococci [4, 18]. In this study representative CXCL8-suppressive streptococci affected significant reductions in NFκB activation in a reporter cell line, indicating one potential immunomodulatory pathway.

For some commensal organisms, the cell components or products that mediate effects on immune and inflammatory responses have been identified and include capsular polysaccharide, short chain fatty acids (SCFA), cellular or secreted proteins/peptides, nucleic acids, flagellin, peptidoglycan and hydrogen peroxide [33–39]. The immunosuppressive mediator produced by *S. salivarius* K12 has not been identified, but two other *S. salivarius* strains secreted a peptide of <3 KDa that mediated immunosuppression [14]. Our experiments using heat and UV-killed bacteria indicated inter-species variability of immunosuppressive mechanism(s). *S. salivarius* and *S. parasanguinis* retained their immunosuppressive abilities after heat killing, implying that the mediator is unlikely to be a secreted metabolite. Conversely, both heat and UV-killing of *S. mitis/oralis* and *Streptococcus* sp*. 2_1_36FAA* isolates ablated their immunosuppressive ability with the result that they caused no significant differences in CXCL8 secretion from both unstimulated and stimulated control cells (Fig. 5). These findings suggest that viable bacterial cells may be essential to immunosuppressive ability in certain strains, indicative of the potential involvement of secreted metabolites, such as SCFA. Inactivation of immunosuppression by UV-killing and not heat-killing indicated the possible involvement of nucleic acids as the mediating factor. Similar mechanisms have previously been observed [40], with high levels of double-stranded RNA in lactic acid bacteria associated with stimulation of interferon-β production, resulting in protective effects in a murine colitis model [41]. An absence of a heat-labile element would suggest that the immunomodulation observed here was not mediated by a protein. Ultimately, the mechanism behind epithelial cell CXCL8 suppression in streptococci appears multifactorial and is potentially strain dependent. Further investigation is required to elucidate the cell machinery conveying this phenomenon. Additionally, multiple mechanisms of modulating host responses may be displayed by one species or organism. For example, the *S. mitis* type strain (CCUG 31611) has been observed to activate an oral epithelial cell aryl hydrocarbon receptor, promoting CXCL8 expression, and to increase transcription of some chemokines in monocytes while also promoting expression of the immunosuppressive cytokine IL-10 [3, 42].

It is not uncommon for similar colonization and survival immune evasion strategies to be employed by commensals and pathogens, and suppression of host inflammatory responses (including secretion of CXCL8) is also induced by the periodontopathogens *P. gingivalis* and *Treponema denticola* [11, 43]. However, *P. gingivalis* utilizes multiple mechanisms targeting a range of cellular and soluble immune defences to cause extensive inhibition of local immune responses, while also activating mechanisms that promote the deregulated inflammatory response, inefficient resolution of inflammation and increased bone resorption associated with periodontitis [44–46]. In contrast, the limiting, immunomodulatory effects of commensals are more subtle, and immunosuppression may be accompanied by properties that promote anti-inflammatory responses and/or enhance cellular homeostasis [4, 47–50].

Many of the 300 presumptive streptococcal isolates in this study caused a modest increase in LDH release from epithelial cells after 24 h co-culture. Hydrogen peroxide and urease are produced by a range of resident oral streptococci and both have been reported to be cytotoxic to epithelial and other cells [51–53]. However, few of our isolates could be defined as cytotoxic in the experimental system employed here. The degree of cell killing may be defined, not only by the strain and the environmental/growth conditions, but also by the m.o.i. and cell type. Moderate toxic effects may be counter-balanced *in vivo* by the effects of these molecules in protecting against acidification and in inhibiting the growth of anaerobes such as *P. gingivalis* and *Prevotella intermedia* [54], as well as other non-oral pathogens [55, 56].

The potential beneficial effects of resident organisms extend beyond immune homeostasis, to include pathogen exclusion, enhancing mucin production and barrier function, induction of antimicrobial host defence peptides, promoting angiogenesis and wound healing [2, 57]. It has been recognized that resident oral bacteria contribute to healthy gingival tissues in that they help to direct the development of oral immune responses and elicit responses that maintain appropriate levels of defensive neutrophils within the gingival epithelium [58, 59]. *S. salivarius* K12, a probiotic isolated from the tongue, down-regulated epithelial cell inflammatory responses and also up-regulated hepcidin [4], a regulator of iron adsorption that activates signalling pathways and reduces the generation of inflammatory markers to lipopolysaccharide [60]. Furthermore, *S. salivarius* K12 stimulated beneficial pathways not considered as pro-inflammatory including type I and II interferon responses and anti-apoptotic responses, and exerted significant effects on the cytoskeleton and adhesive properties of the host cells [4]. An appropriate level of commensals capable of exhibiting a combination of immunomodulatory and homeostatic properties may be essential for maintaining oral health, and studies into the potentially beneficial capabilities of our isolates are continuing. Understanding the processes that underlie the maintenance of healthy oral tissues will help to develop preventive strategies and aid in the identification of potential probiotic organisms. These could also lead to strategies to promote oral health, maintaining the balance of perpetual fluctuations required for oral allostasis [61, 62].

## Supplementary Data

Supplementary material 1

## References

[1] Huttenhower C, Gevers D, Knight R, Abubucker S, Badger JH (2012). Structure, function and diversity of the healthy human microbiome. Nature.

[2] Devine DA, Marsh PD, Meade J (2015). Modulation of host responses by oral commensal bacteria. J Oral Microbiol.

[3] Engen SA, Rørvik GH, Schreurs O, Blix IJ, Schenck K (2017). The oral commensal *Streptococcus mitis* activates the aryl hydrocarbon receptor in human oral epithelial cells. Int J Oral Sci.

[4] Cosseau C, Devine DA, Dullaghan E, Gardy JL, Chikatamarla A (2008). The commensal *Streptococcus salivarius* K12 downregulates the innate immune responses of human epithelial cells and promotes host-microbe homeostasis. Infect Immun.

[5] Ivanov II, Honda K (2012). Intestinal commensal microbes as immune modulators. Cell Host Microbe.

[6] Belkaid Y, Naik S (2013). Compartmentalized and systemic control of tissue immunity by commensals. Nat Immunol.

[7] Round JL, Mazmanian SK (2009). The gut microbiota shapes intestinal immune responses during health and disease. Nat Rev Immunol.

[8] Neish AS (2009). Microbes in gastrointestinal health and disease. Gastroenterol.

[9] Hooper LV (2009). Do symbiotic bacteria subvert host immunity?. Nat Rev Microbiol.

[10] Morgan XC, Segata N, Huttenhower C (2013). Biodiversity and functional genomics in the human microbiome. Trends Genet.

[11] Darveau RP (2010). Periodontitis: a polymicrobial disruption of host homeostasis. Nat Rev Microbiol.

[12] Graves D (2008). Cytokines that promote periodontal tissue destruction. J Periodontol.

[13] Hasegawa Y, Mans JJ, Mao S, Lopez MC, Baker HV (2007). Gingival epithelial cell transcriptional responses to commensal and opportunistic oral microbial species. Infect Immun.

[14] Kaci G, Lakhdari O, Dore J, Ehrlich SD, Renault P (2011). Inhibition of the NF-kappa B pathway in human intestinal epithelial cells by commensal *Streptococcus salivarius*. Appl Environ Microbiol.

[15] Kaci G, Goudercourt D, Dennin V, Pot B, Dore J (2014). Anti-inflammatory properties of *Streptococcus salivarius*, a commensal bacterium of the oral cavity and digestive tract. Appl Environ Microbiol.

[16] Sliepen I, Van Damme J, Van Essche M, Loozen G, Quirynen M (2009). Microbial interactions influence inflammatory host cell responses. J Dent Res.

[17] Guglielmetti S, Taverniti V, Minuzzo M, Arioli S, Stuknyte M (2010). Oral bacteria as potential probiotics for the pharyngeal mucosa. Appl Environ Microbiol.

[18] Zhang G, Chen R, Rudney JD (2011). *Streptococcus cristatus* modulates the *Fusobacterium nucleatum*-induced epithelial interleukin-8 response through the nuclear factor-kappa B pathway. J Periodontol Res.

[19] Hoover CI, Newbrun E (1977). Survival of bacteria from human dental plaque under various transport conditions. J Clin Microbiol.

[20] Jang H-J, Kang M-S, S-H Y, Hong J-Y, Hong S-P (2016). Comparative study on the characteristics of *Weissella cibaria* Cmu and probiotic strains for oral care. Molecules.

[21] Kang M-S, J-S O, Lim H-S, Kim S-M, Lee H-C (2011). Effect of *Weissella cibaria* on *Fusobacterium nucleatum*-induced interleukin-6 and interleukin-8 production in KB cells. J Bacteriol Virol.

[22] Kolenbrander PE, Palmer RJ, Rickard AH, Jakubovics NS, Chalmers NI (2006). Bacterial interactions and successions during plaque development. Periodontology.

[23] Aas JA, Paster BJ, Stokes LN, Olsen I, Dewhirst FE (2005). Defining the normal bacterial flora of the oral cavity. J Clin Microbiol.

[24] Peterson SN, Snesrud E, Liu J, Ong AC, Kilian M (2013). The dental plaque microbiome in health and disease. Plos One.

[25] Kilian M, Frandsen EVG, Haubek D, Poulsen K (2006). The etiology of periodontal disease revisited by population genetic analysis. Periodontology.

[26] Belda-Ferre P, Alcaraz LD, Cabrera-Rubio R, Romero H, Simon-Soro A (2012). The oral metagenome in health and disease. Isme Journal.

[27] Mitchell J (2011). *Streptococcus mitis*: walking the line between commensalism and pathogenesis. Molecular Oral Microbiology.

[28] Di Pierro F, Risso P, Poggi E, Timitilli A, Bolloli S (2018). Use of *Streptococcus salivarius* K12 to reduce the incidence of pharyngo-tonsillitis and acute otitis media in children: a retrospective analysis in not-recurrent pediatric subjects. Minerva Pediatrica.

[29] Twetman S, Derawi B, Keller M, Ekstrand K, Yucel-Lindberg T (2009). Short-term effect of chewing gums containing probiotic *Lactobacillus reuteri* on the levels of inflammatory mediators in gingival crevicular fluid. Acta Odontol Scand.

[30] Belibasakis GN, Thurnheer T, Bostanci N (2013). Interleukin-8 responses of multi-layer gingival epithelia to subgingival biofilms: role of the "red complex" species. PLoS One.

[31] Peyyala R, Kirakodu SS, Novak KF, Ebersole JL (2013). Oral epithelial cell responses to multispecies microbial biofilms. J Dent Res.

[32] Rocha CS, Lakhdari O, Blottiere HM, Blugeon S, Sokol H (2012). Anti-inflammatory properties of dairy lactobacilli. Inflamm Bowel dis.

[33] Kamada N, Nunez G (2014). Regulation of the immune system by the resident intestinal bacteria. Gastroenterol.

[34] Bienenstock J, Gibson G, Walker WA, Neish AS (2013). New insights into probiotic mechanisms: a harvest from functional and metagenomic studies. Gut Microbes.

[35] Brestoff JR, Artis D (2013). Commensal bacteria at the interface of host metabolism and the immune system. Nature Immunol.

[36] Chang PV, Hao L, Offermanns S, Medzhitov R (2014). The microbial metabolite butyrate regulates intestinal macrophage function via histone deacetylase inhibition. Proc Natl Acad Sci USA.

[37] Atarashi K, Tanoue T, Shima T, Imaoka A, Kuwahara T (2011). Induction of colonic regulatory T cells by indigenous *Clostridium* Species. Science.

[38] Hevia A, Delgado S, Sánchez B, Margolles A (2015). Molecular players involved in the interaction between beneficial bacteria and the immune system. Front Microbiol.

[39] Erttmann SF, Gekara NO (2019). Hydrogen peroxide release by bacteria suppresses inflammasome-dependent innate immunity. Nat Commun.

[40] Powell DA, Ma M, So M, Frelinger JA (2018). The commensal *Neisseria musculi* modulates host innate immunity to promote oral colonization. ImmunoHorizons.

[41] Kawashima T, Kosaka A, Yan H, Guo Z, Uchiyama R (2013). Double-Stranded RNA of intestinal commensal but not pathogenic bacteria triggers production of protective interferon-β. Immunity.

[42] Engen SA, Schreurs O, Petersen F, Blix IJS, Baekkevold ES (2018). The regulatory role of the oral commensal *Streptococcus mitis* on human monocytes. Scand J Immunol.

[43] Hajishengallis G, Lamont RJ (2014). Breaking bad: manipulation of the host response by *Porphyromonas gingivalis*. Eur J Immunol.

[44] Arjunan P, Meghil MM, Pi W, Xu J, Lang L (2018). Oral pathobiont activates anti-apoptotic pathway, promoting both immune suppression and oncogenic cell proliferation. Scientific Reports.

[45] Curtis MA, Diaz PI, Van Dyke TE (2020). The role of the microbiota in periodontal disease. Periodontology.

[46] Hajishengallis G (2015). Periodontitis: from microbial immune subversion to systemic inflammation. Nat Rev Immunol.

[47] Curtis MA, Zenobia C, Darveau RP (2011). The relationship of the oral microbiotia to periodontal health and disease. Cell Host & Microbe.

[48] Hajishengallis G (2011). Immune evasion strategies of Porphyromonas gingivalis. J Oral Biosci.

[49] Zenobia C, Luo XL, Hashim A, Abe T, Jin L (2013). Commensal bacteria-dependent select expression of CXCL2 contributes to periodontal tissue homeostasis. Cell Microbiol.

[50] Olsen I, Taubman MA, Singhrao SK (2016). *Porphyromonas gingivalis* suppresses adaptive immunity in periodontitis, atherosclerosis, and Alzheimer's disease. J Oral Microbiol.

[51] Okahashi N, Sumitomo T, Nakata M, Sakurai A, Kuwata H (2014). Hydrogen peroxide contributes to the epithelial cell death induced by the oral mitis group of streptococci. Plos One.

[52] Okahashi N, Nakata M, Kuwata H, Kawabata S (2016). *Streptococcus oralis* induces lysosomal impairment of macrophages via bacterial hydrogen peroxide. Infection and Immunity.

[53] Mora D, Arioli S (2014). Microbial urease in health and disease. PLoS Pathog.

[54] Herrero ER, Slomka V, Bernaerts K, Boon N, Hernandez-Sanabria E (2016). Antimicrobial effects of commensal oral species are regulated by environmental factors. J Dent.

[55] Scoffield JA, Wu H (2015). Oral streptococci and nitrite-mediated interference of *Pseudomonas aeruginosa*. Infect Immun.

[56] Whiley RA, Fleming EV, Makhija R, Waite RD (2015). Environment and colonisation sequence are key parameters driving cooperation and competition between *Pseudomonas aeruginosa* cystic fibrosis strains and oral commensal streptococci. Plos One.

[57] Kumar PS, Mason MR (2015). Mouthguards: does the indigenous microbiome play a role in maintaining oral health?. Front Cell Infect Microbiol.

[58] Dixon DR, Bainbridge BW, Darveau RP (2004). Modulation of the innate immune response within the periodontium. Periodontology.

[59] Dixon DR, Reife RA, Cebra JJ, Darveau RP (2004). Commensal bacteria influence innate status within gingival tissues: a pilot study. J Periodontol.

[60] Domenico D, Zhang TY, Koening CL, Branch RW, London N (2010). Hepcidin mediates transcriptional changes that modulate acute cytokine-induced inflammatory responses in mice. J Clin Invest.

[61] Zaura E, Jacob M (2015). Towards understanding oral health. Caries research.

[62] Marsh PD, Head DA, Devine DA (2015). Dental plaque as a biofilm and a microbial community—Implications for treatment. J Oral Biosci.

