# Supplementary material for: Immunomodulatory streptococci that inhibit CXCL8 secretion and NFκB activation are common members of the oral microbiota
Source: J Med Microbiol. 2021 Mar 18;70(3):001329. doi: 10.1099/jmm.0.001329 (PMC8346732; doi:10.1099/jmm.0.001329)
Supplement: Supplementary material 1 [file jmm-70-329-s001.pdf]

Supplementary Material - Immunomodulatory streptococci that inhibit CXCL8 secretion and NFκB activation are common members of the oral microbiota.

Sarah Myers, Thuy Do, Josephine L. Meade, Aradhna Tugnait, Jon J. Vernon, Jelena Pistolic, Robert E. W. Hancock, Philip D. Marsh, Harsh M. Trivedi, Dandan Chen, Deirdre A. Devine

Supplementary Table 1: Viable bacterial counts from the plaque of orally healthy subjects and subjects with gingivitis.

There were no statistically significant differences in counts comparing supragingival plaque from the healthy group compared with the gingivitis group (Mann Whitney u test). Subgingival plaque from the gingivitis group had similar total anaerobic and presumptive Actinomyces counts compared with supragingival plaque samples from the same individuals, but the total facultative counts were significantly higher (p<0.01; Wilcoxon signed rank test) in supragingival compared with subgingival plaque.

*¥ - log_{10} cfu mL^{-1}: colony forming units per mL, n/a - Not applicable, € - AnO_2: Anaerobic total count, † - CO_2: Facultative total count, # - MSA: Streptococci grown on Mitis Salivarius agar, § - Cadmium fluoride acriflavin tellurite agar (CFAT) plates for enumeration of Actinomyces spp. were incubated anaerobically for two days. CFAT contained per L distilled water: Trypticase soy broth 30 g, glucose 5 g, agar 15 g, cadmium sulphate 13 mg, sodium fluoride 80 mg, neutral acriflavin 1.2 mg, potassium tellurite 2.5 mg, basic fuchsine 0.25 mg and defibrinated horse blood 50 mL; adjusted to pH 7.3, ** - p < 0.01 comparing counts in supragingival and subgingival plaque (gingivitis), SD – Standard deviation.*

Subject	***Healthy/ Gingivitis***	***Supragingival plaque log_{10} cfu mL^{-1}¥***				***Streptococci as % of* total viable count**	***Subgingival plaque log_{10} cfu mL^{-1}¥***				***Streptococci as % of* total viable count**
		**AnO_2€**	**CO_2†**	**MSA#**	**CFAT§**		**AnO_2**	**CO_2**	**MSA**	**CFAT**	
**H1**	Healthy	6.6	6.1	5.8	6.1	18.4	n/a	n/a	n/a	n/a	n/a
**H2**	Healthy	5.8	6.2	5.9	6.0	55.1	n/a	n/a	n/a	n/a	n/a
**H3**	Healthy	5.5	6. 1	5.9	5.8	60.0	n/a	n/a	n/a	n/a	n/a
**H4**	Healthy	6.5	6.1	5.8	5.7	23.6	n/a	n/a	n/a	n/a	n/a
**H5**	Healthy	5.5	6.5	5.8	5.5	20.6	n/a	n/a	n/a	n/a	n/a
Mean (SD)		***6.0 (0.5)***	***6.2 (0.2)***	***5.9 (0.0)***	***5.8 (0.2)***	***35.6 (20.2)***					
**G1**	Gingivitis	6.2	6.2	6.5	5.6	100.0	6.4	5.4	6.3	5.5	79.2
**G2**	Gingivitis	7.3	6.3	6.3	6.0	11.1	6.58	5.8	6.0	5.7	24.2
**G3**	Gingivitis	6.7	6.3	5.9	5.9	17.4	6.2	5.8	5.9	5.4	41.3
**G4**	Gingivitis	6.5	5.6	5.3	5.3	5.7	6.3	5.3	4.9	5.5	4.6
**G5**	Gingivitis	5.6	6.0	5.6	5.6	39.2	5.5	5.6	5.3	5.2	46.5
Mean (SD)		***6.5 (0.6)***	*****6.1 (0.3)***	***5.9 (0.5)***	***5.7 (0.3)***	***34.7 (38.7)***	***6.4 (0.2)***	*****5.6 (0.3)***	***5.7 (0.6)***	***5.5 (0.2)***	***39.2 (27.7)***

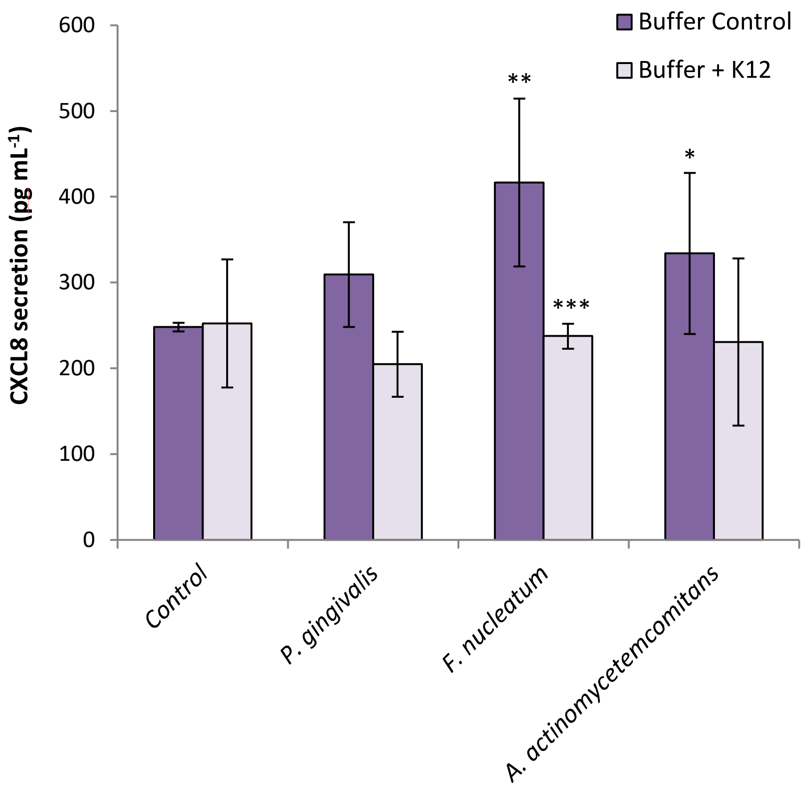

Supplementary Figure 1: *S. salivarius* K12 Inhibition of CXCL8 secretion by dysplastic human oral keratinocyte (DOK) cells in response to oral bacteria.

Dark bars – additions to cells: Buffer - no K12 (Control), Porphyromonas gingivalis W50, Fusobacterium nucleatum ATCC 10953, Aggregatibacter actinomycetemcomitans NCTC 9710. Light bars – additions to cells: S. salivarius K12 plus buffer (Control), K12 plus P. gingivalis W50, K12 plus F. nucleatum ATCC 10953, K12 plus A. actinomycetemcomitans NCTC 9710.
** - p <0.05 and ** - p <0.01, compared with buffer Control. *** - p <0.001 comparing F. nucleatum alone with F. nucleatum plus K12. Error bars represent ± standard error of the mean.*

DOK cells (European Collection of Cell Cultures; ECACC) were grown in cell culture flasks (Sarstedt) at 37°C in a humidified 5% CO_2 incubator in Dulbecco's modified Eagles' medium; (DMEM, Sigma) plus 10 % (v/v) foetal calf serum (FCS; Harlam Sera Labs, UK), 2 mM L-glutamine (Sigma) and 5 µg mL^{-1} hydrocortisone (Sigma, UK). Cells were passaged using trypsin-EDTA (Sigma) and washed twice post detachment with the same complete DMEM medium. Cells were used between passage number two and 20. They were seeded in 24 well plates (Sarstedt) at a density of $1x10^5$ cells/well and grown for 48 hours at 37°C in 5% CO_2 in complete DMEM. Complete DMEM was removed from the monolayers (ca. 95% confluent) and replaced by serum-free DMEM; following two hours incubation the monolayers were co-incubated with bacteria at a MOI of 50:1.

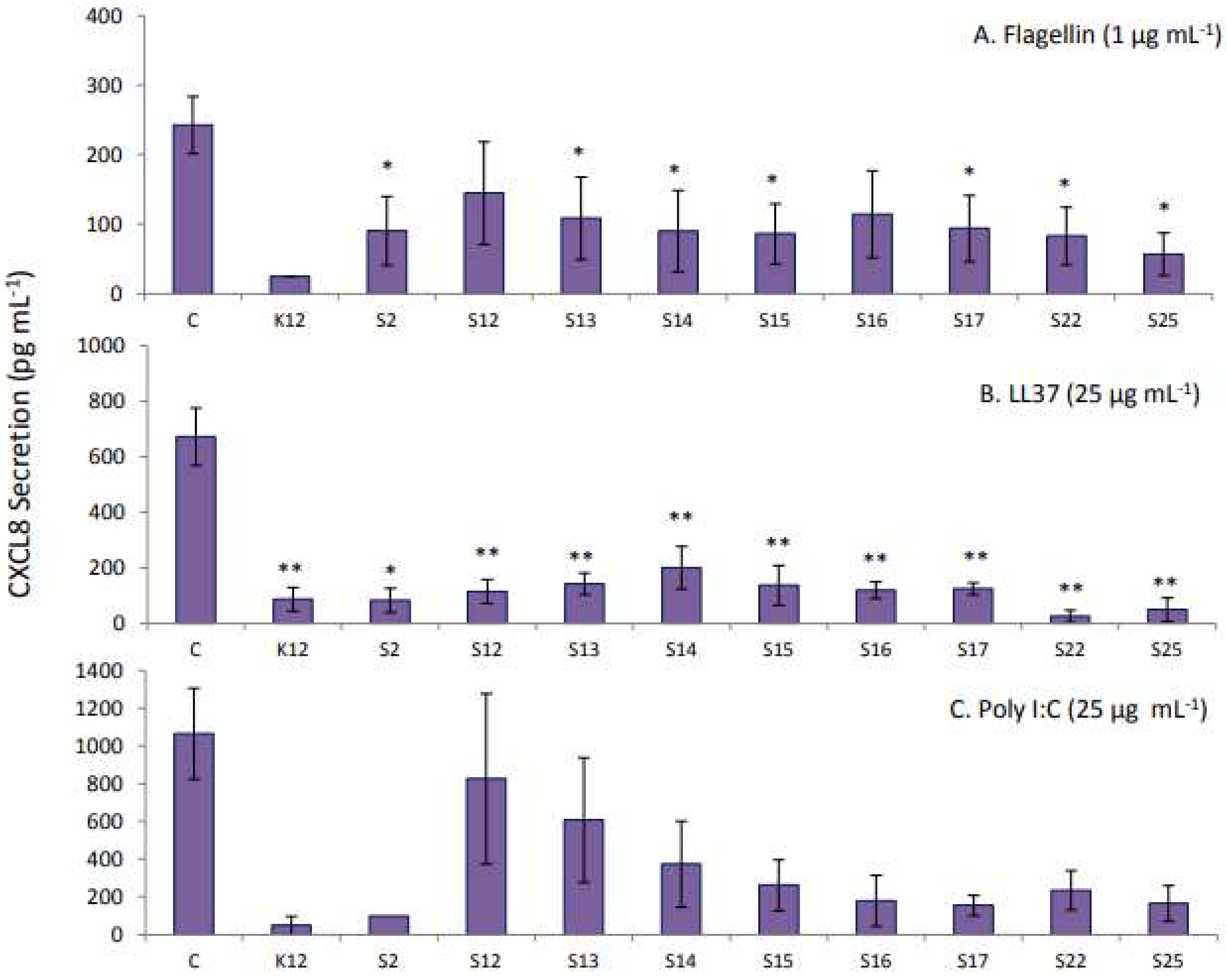

Supplementary Figure 2: CXCL8 secretion (pg mL^{-1}) by 16HBE14o- cells incubated with streptococci from the tongue, stimulated with A – flagellin (1 µg mL^{-1}), B – LL-37 (25 µg mL^{-1}) or C – Poly I:C (25 µg mL^{-1}).

*C – control with no streptococci. K12, S2, S12, S13, S14, S15, S16 & S17 – S. salivarius. S22 & S25 – S. parasanguinis. Error bars represent ± standard error of the mean. Statistical comparisons of streptococci co-cultures versus control CXCL8 secretion were performed using Dunn's Multiple Comparison Test and a Kruskal-Wallis post-test; **p<0.01, * p<0.05. Poly I:C experimental data represents duplicate data and no statistical significance was therefore calculated.*

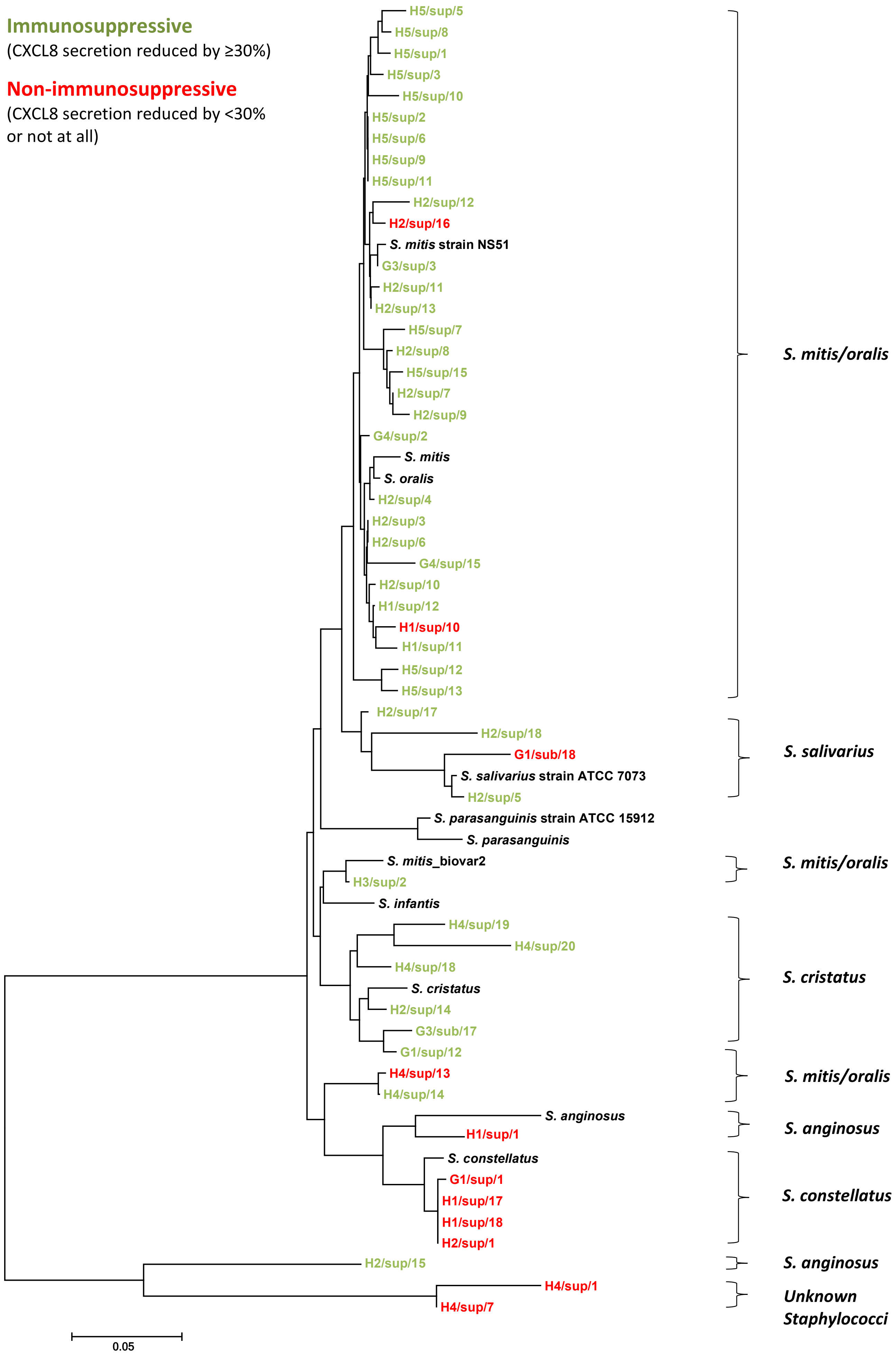

Immunosuppressive
(CXCL8 secretion reduced by ≥30%)
Non-immunosuppressive
(CXCL8 secretion reduced by <30%
or not at all)
H5/sup/5
H5/sup/8
H5/sup/1
H5/sup/3
H5/sup/10
H5/sup/2
H5/sup/6
H5/sup/9
H5/sup/11
H2/sup/12
H2/sup/16
S. mitis strain NS51
G3/sup/3
H2/sup/11
H2/sup/13
H5/sup/7
H2/sup/8
H5/sup/15
H2/sup/7
H2/sup/9
G4/sup/2
S. mitis
S. oralis
H2/sup/4
H2/sup/3
H2/sup/6
G4/sup/15
H2/sup/10
H1/sup/12
H1/sup/10
H1/sup/11
H5/sup/12
H5/sup/13
S. mitis/oralis
H2/sup/17
H2/sup/18
G1/sub/18
S. salivarius strain ATCC 7073
H2/sup/5
S. salivarius
S. parasanguinis strain ATCC 15912
S. parasanguinis
S. mitis_biovar2
H3/sup/2
S. mitis/oralis
S. infantis
H4/sup/19
H4/sup/20
H4/sup/18
S. cristatus
H2/sup/14
G3/sub/17
G1/sup/12
S. cristatus
H4/sup/13
H4/sup/14
S. mitis/oralis
S. anginosus
H1/sup/1
S. anginosus
S. constellatus
G1/sup/1
H1/sup/17
H1/sup/18
H2/sup/1
S. constellatus
H2/sup/15
S. anginosus
H4/sup/1
H4/sup/7
Unknown Staphylococci
0.05

Supplementary Figure 3: Dendrogram representing the phylogenetic relationship between partial 16S rRNA gene sequences (amplified using universal primers 27f and 1492r, and sequenced using 519r) of 51 streptococci isolated from the dental plaque of healthy and gingivitis subjects and 11 known streptococcus spp. strains. *Phylograms were constructed using the Neighbour-Joining algorithm in MEGA v.5 (http://www.megasoftware.net).*

Green labels represent immunosuppressive isolates (demonstrating a reduction in CXCL8 secretion ≥30%), whilst red labels represent non-immunosuppressive isolates (demonstrating a reduction in CXCL8 secretion <30%, or no reduction at all). Black labels represent the known, control strains sequenced and the identities of the isolates.
